# Re-purposing Chloroquine for Glioblastoma: Potential Merits and Confounding Variables

**DOI:** 10.3389/fonc.2018.00335

**Published:** 2018-08-27

**Authors:** Patrick Weyerhäuser, Sven R. Kantelhardt, Ella L. Kim

**Affiliations:** ^1^Institute of Cancer Therapeutics, University of Bradford, Bradford, United Kingdom; ^2^Clinic for Neurosurgery, Johannes Gutenberg University Medical Center Mainz, Mainz, Germany; ^3^Laboratory for Experimental Neurooncology, Clinic for Neurosurgery, Johannes Gutenberg University Medical Center Mainz, Mainz, Germany

**Keywords:** glioblastoma, chloroquine, radio-sensitization, autophagy, glioma stem-like cells

## Abstract

There is a growing evidence that antimalarial chloroquine could be re-purposed for cancer treatment. A dozen of clinical trials have been initiated within the past 10 years to test the potential of chloroquine as an adjuvant treatment for therapy–refractory cancers including glioblastoma, one of the most aggressive human cancers. While there is considerable evidence for the efficacy and safety of chloroquine the mechanisms underlying the tumor suppressive actions of this drug remain elusive. Up until recently, inhibition of the late stage of autophagy was thought to be the major mechanism of chloroquine-mediated cancer cells death. However, recent research provided compelling evidence that autophagy-inhibiting activities of chloroquine are dispensable for its ability to suppress tumor cells growth. These unexpected findings necessitate a further elucidation of the molecular mechanisms that are essential for anti-cancer activities of CHQ. This review discusses the versatile actions of chloroquine in cancer cells with particular focus on glioma cells.

## Introduction

Glioblastoma (GB) is one of the most lethal human cancers (1). Despite its rarity, GB is among the top priorities in clinical oncology due to its extremely aggressive pattern, high mortality rate and unsatisfactory efficacy of current treatments. An eventual mortality rate close to 100%, 5–years survival rate of <10%, and a median survival of only 15 months remain unimproved since the establishment of standard frontline therapy for GB in 2005 (2, 3). The current standard of care for GB is based on the “one-treatment-for-all” principle and consists of a surgical resection as complete as feasible, followed by combined treatment with hypofractionated radiation therapy and non-selective chemotherapy with DNA alkylating agent temozolomide (TMZ) followed by six cycles of chemotherapy alone (3). However, the clinical effectiveness of TMZ is rather moderate (survival benefit of 2 months compared with radiotherapy alone) and restricted to a subset of GBs (~50%) lacking methyl-guanine-methyl-transferase (MGMT), an enzyme that removes the alkyl group from TMZ-induced O6-methylguanine DNA adducts (4). GBs re-grow inevitably after (or under) radio-chemotherapy. For recurrent GBs, there is no generally accepted standard therapy. None of the experimentally tested therapeutic options led to significant survival benefit (5). Post-treatment recurrence due to intrinsic and acquired resistance to cytotoxic treatments pose the major challenge to effective treatments of GB. The hallmark of GB's genetic landscape is the co-occurrence of multiple defects in key cancer-related pathways that use distinct mechanisms yet have partially overlapping functions. RTK, pRb and p53 have been identified as core pathways impaired nearly universally in the majority of GBs (6). Multiplicity of genetic aberrations affecting different pathways in conjunction with the functional redundancy of affected pathways poses a challenge for mono-targeted therapies for GB. Adding a further level of complexity, there is considerable heterogeneity of cell types constituting GBs.

Development of multi-targeted therapeutic approaches using a combination of drugs or a drug with a broad spectrum of targets might provide the solution to overcome intrinsic and acquired resistance of GBs to cytotoxic treatments.

## Chloroquine: a convoluted path from malaria to cancer treatment

Chloroquine (CHQ) is a well-known antimalarial that has recently attracted considerable attention for its anti-neoplastic activites. Application of CHQ for cancer treatment is an example of drug re-purposing, a strategy for identifying new therapeutic indications for drugs that have initially been developed for different medical applications (7). Synthetized at I.G. Farbenindustrie Bayer A.G. Laboratories (Elberfeld, Germany) in 1934, CHQ has been the drug of choice for malaria treatment for several decades till its role as anti–malarial has diminished due to the emergence of CHQ–resistant strains of the malaria parasite. One of the early encounters of anti-neoplastic effects of CHQ have been made during an anti-malaria trial launched by WHO in North Africa in the 1970's. It was noticed that the incidence of Burkitt's lymphoma dropped profoundly in the CHQ-treated population during the trial but returned to the basal level after the trial has been discontinued (8). This unexpected observation has remained unfollowed until a series of experimental studies reported on anti–neoplastic effect of CHQ in different types of cancer cells (9). In particular, the potential of CHQ to sensitize neoplastic cells to radiation and some other types of chemotherapy has been emerging as an approach to target treatment-refractory cancers including GBs. Currently, 17 clinical studies have been initiated to test the effects of CHQ as adjuvant treatment for different types of cancer including GB (Table 1) (12). Interest to CHQ as an adjuvant treatment for GB was sparked by the initial observation that addition of CHQ to standard therapy leads to a significant prolongation of survival in patients with GB (17) (10). After the initial demonstration that CHQ potentiates therapeutic effects of standard therapy in a double-blinded clinical trial (Phase III) involving a cohort of 30 patients with newly diagnosed GB, (10) further encouraging results have been reported in a case study with 5 patients suffering from recGB treated with CHQ and re-irradiation (18). These observations are coherent with the results from experimental studies indicating that chloroquine can potentiate cytotoxicity of TMZ and ionizing radiation in glioma cells (19–22).

**Table 1:** Summary of clinical trials testing chloroquine in GBs.

**Study ID**	**Phase**	**Patient group**		**Treatment**	**Outcomes**
		**Age**	**Diagnosis**		
NCT00224978	III	18–65	First/second recurrent or relapsed GB (WHO stage = IV) in one hemisphere	Carmustine + radiotherapy + placebo vs. Carmustine + radiotherapy + chloroquine	- Increase OS from 11 to 24 months - No statistical significance - Well tolerated (10, 11)
NCT03243461	III	3–18	Untreated pediatric high-grade glioma (WHO stage ≥ III)	Temozolomide + radiotherapy + valproic acid vs. Temozolomide + radiotherapy + chloroquine	*Estimated study start: Feb. 2018* (12, 13)
NCT02432417	II	18–70	Newly diagnosed IDH wild-type GB (WHO stage = IV)	Radiotherapy + chloroquine	*Estimated study start: Jan. 2020* (12, 14)
NCT02378532	I	≥ 18	Newly diagnosed GB (WHO stage = IV) and confirmed MGMT and EGFRvIII status	Temozolomide + Radiotherapy + chloroquine	*Currently recruiting* (12, 15)
NCT01727531		≥18	Solid primary tumor and at least one brain metastasis	Whole-brain radiotherapy + chloroquine	*No results published* 16

CHQ (7-chloro-4-(4-diethylamino-1-methylbutylamino)-quinoline) is a small, lipophilic, amphiphilic and weakly basic tertiary amine with pK_a_s of 8.4 and 10.2 (12, 23). At the physiological pH of 7.4, CHQ is unprotonated and highly membrane-penetrating (12). Once inside the cell, CHQ accumulates in acidic compartments and becomes protonated. As a consequence, it raises the intra–organellar pH and affects the activity of endosomes, lysosomes, autophagosomes, and autophagolysosomes (23). Owing to its lysosomotropic properties, CHQ accumulates primarily in the lysosome, where the increase of the lysosomal pH leads to a blockage of the lysosome-autophagosome fusion, a critical event during the late stage of autophagy (24). Good solubility and rapid absorption are attractive pharmacological properties of CHQ. It is rapidly absorbed when administered orally, but sub cutaneous, intra muscular, and rectal administrations are likewise possible (25).

CHQ can elicit an array of distinct biological responses in the CNS, depending on the dose and cell type. The lowest threshold of CHQ concentrations to induce neuronal death *in vitro* is around 20 μM (26, 27). Similar values for cytotoxic concentrations of CHQ were found in normal astrocytes (28) or neoplastic cells derived from astrocytic tumors (29, 30). However, at concentrations of 10 μM or lower, CHQ elicits neuroprotective effects in the context of oxidative damage (31). Thus, various functional outcomes can be elicited by CHQ depending on the cell type, particular pathophysiological condition, dose of the drug and treatment context. While there is an abundance of information about safety and tolerability profiles of CHQ in the context of non–cancer pathologies, CHQ application for cancer treatment will require establishing tolerability ranges in cancer patients and at cancer-relevant doses. This consideration is of special importance in the context of brain tumors, which are protected by the blood brain barrier. A phase I/II trial addressing the effects and feasibility of escalating CHQ doses for GB treatment found that CHQ doses used for treating rheumatoid arthritis may not be sufficient to effectively inhibit autophagy when used in combination with TMZ and radiation in patients with GB (32).

## Molecular mechanisms of anti-neoplastic activities of chloroquine

The mechanisms of radio- or chemo sensitization mediated by CHQ in glioma cells are not entirely understood. Modulation of the autophagic response is by far the most intensively investigated mechanism of CHQ in non-neoplastic and cancer cells. Until recently, the generally accepted view was that inhibition of autophagy is the major route of cancer cell death induced by CHQ (33). Indeed, several lines of experimental evidence suggest the importance of autophagic inhibition as the underlying mechanisms of radio-sensitization by CHQ. Knock down of beclin-1 or pharmacological inhibition of autophagy by 3-methyladenine or interference with autophagy-promoting signaling mediated through the PI3K/Akt (20) or EGFR signaling (34) have been shown to impair the radio/chemo-sensitizing ability of CHQ in glioma cells. However, the seemingly well delineated causative relationship between CHQ effects on autophagy and tumor suppression has recently been challenged by some very surprising findings coming from the pharmaceutical oncology field. Nearly simultaneously, research teams from AstraZeneka, Novartis and Pfizer have provided compelling evidence that tumor–suppressing effects of CHQ are independent from its autophagy-inhibiting activities (35, 36). Intriguingly, CHQ-induced cell death was found to be related with the inhibition of cholesterol biosynthesis by autophagy-related pathways but not with autophagy inhibition *per se* (36). These findings prompt to hypothesize that modulation of the cell metabolism might be one of the mechanisms underlying the anti-neoplastic efficacy of CHQ, which affects a range of metabolic processes including the amino acid metabolism, (37) glucose metabolism (38) and mitochondrial metabolism (39). Interestingly, CHQ potently inhibits glyconeogenesis, (40) which is a compensatory mechanism supporting the survival of cancer cells bearing mutations in the isocitrate dehydrogenase (*IDH*) gene. *IDH1/2* genes code for metabolic enzymes that interconvert isocitrate and α-ketoglutarate. Loss of catalytic activity caused by point mutations in *IDH1/2* genes leads to a decrease in α-ketoglutarate and increased production of *D–*2–hydroxyglutarate (41, 42). In glial tumors, *IDH1/2* mutational status is regarded as one of the most important diagnostic and prognostic biomarkers (43, 44). Point mutations in *IDH1/2* associate with longer survival and are found in about 80% of anaplastic astrocytoma (WHO Grade III) and secondary GBs (GBs that progress from lower grade gliomas), but only rarely (< 10%) in primary GBs (GBs that occur without precursor lesions). Although the relationship between *IDH1/2* mutational status and sensitivity to CHQ in gliomas remains to be established, the recently proposed hypothesis that *IDH1/2* mutations might be predictive of the efficacy of CHQ in gliomas seems plausible (42). Recently launched clinical studies aiming to validate the association between *IDH1/2*-mutated molecular subtype and sensitivity to CHQ will test this hypothesis (45).

## Functional pleiotropy of chloroquine: the balance of good and evil

The diversity of CHQ effects reflects the functional pleiotropy of its molecular targets, which include multi-functional factors as transcription factor NF-κB, (46) or DNA damage-inducible factors like the ataxia telangiectasia mutated (ATM) kinase (47) and its downstream target tumor suppressor p53 (48). A broad versatility of responses that can be mediated by CHQ can be exemplified by its effects on p53 whose functional status is an important factor determining the ultimate outcome from CHQ treatment in cancer cells. This, in fact, is not surprising considering the nodal position of p53 in several regulatory hubs that govern diverse cellular responses to different types of stress (49, 50). The ability to trigger distinct effects such as cell survival or cell death is the key fundamental of p53 function as the “guardian of the genome” (51). Amidst a great multitude of factors influencing the choice between pro-survival and death-promoting activities of p53, (52) the ability to repair DNA damages is essential for promoting cell survival after cell injury. Activation of p53 signaling upon DNA damage can lead to a transient arrest of the cell cycle, enabling DNA repair, or cell death, if the extent of DNA damage exceeds the repair capacity of the cell. Whereas the ability of CHQ to induce p53-dependent apoptosis has been well-documented (22, 27, 29), the mechanism of p53 activation by CHQ remains elusive. In the canonical DNA damage response (DDR), activation of the ATM/Chk1/p53 signaling is the initial event in a signaling cascade triggered by DNA-double strand breaks (53). However, CHQ does not cause direct DNA damage. It has been proposed that topological perturbations in the chromatin structure caused by CHQ intercalation into the DNA helix (54–57) may be sensed by ATM leading to its activation by autophosphorylation (47). Alternatively (or in addition) to its direct effects on DNA topology, CHQ can cause DNA breakage through an indirect mechanism involving mitochondrial damage (58). Considering that both ATM and p53 are sensitive to oxidative stress, (59, 60) these findings indicate that activation of the ATM-p53 signaling by CHQ might be triggered by oxidative DNA damage. Interestingly, while activating key mediators of DDR, CHQ has an intrinsic repair-inhibiting activity manifest in different types of normal and neoplastic cells *in vitro* (30, 58) and *in vivo* (61). Although the exact mechanisms of CHQ–mediated inhibition of DNA repair remain unknown, they are likely to reflect the causative relationship between impaired autophagy and deficient DNA repair (62). It is tempting to hypothesize that conflicting signals generated through the dual ability of CHQ to activate key mediators of DDR and to suppress DNA repair, play a role in shifting the balance in favor of cell death. Potentially conflicting signals can also emanate from the p53 transcriptional response induced by CHQ. p53 activation leads to transcriptional up-regulation of Bax1, which is indispensable for CHQ–induced apoptosis, (27) but also induces a battery of genes that promote cell survival through the activation of autophagic response (52).

**Figure 1:**
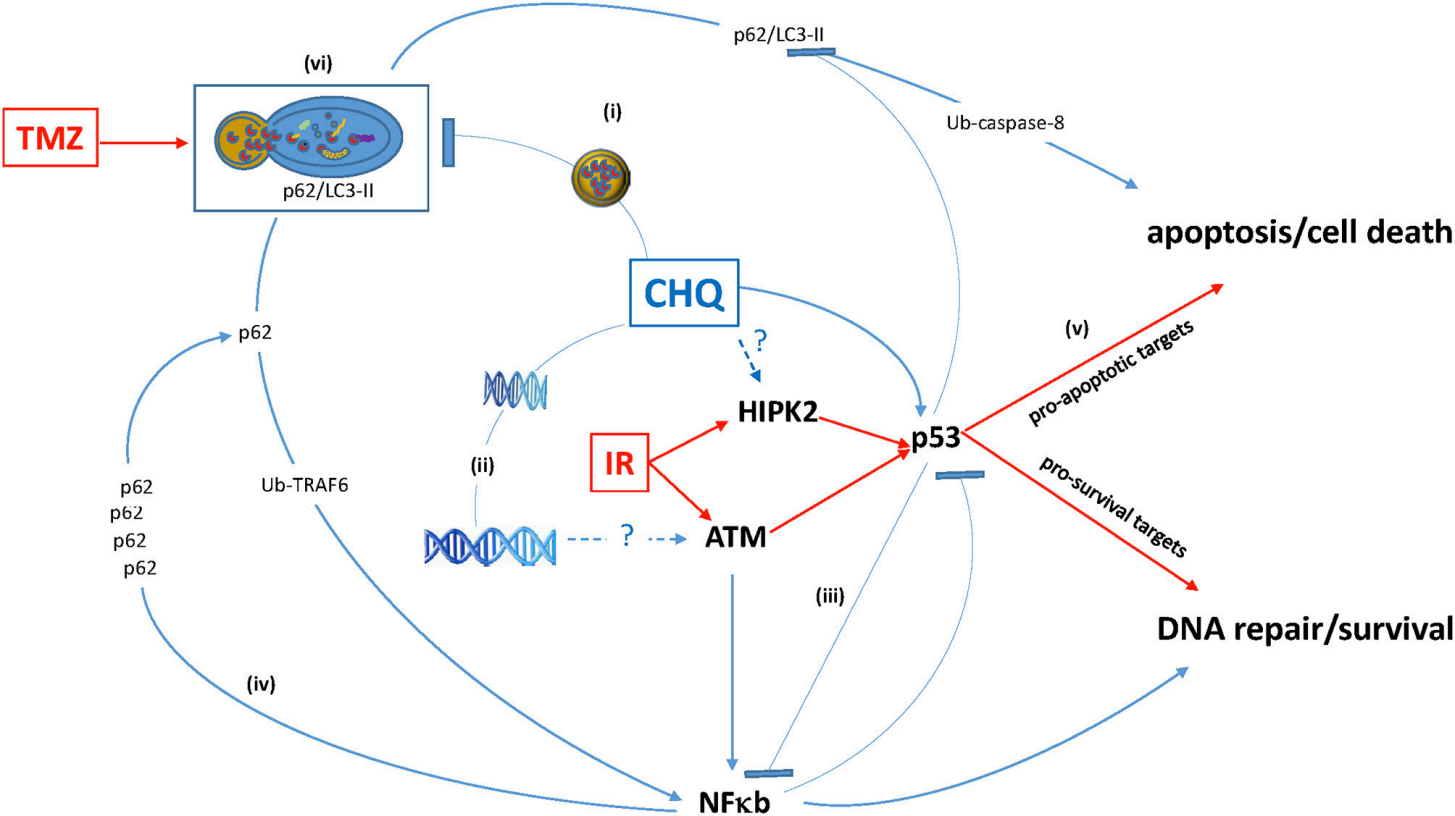
Antagonistic pleiotropy of multifunctional hub proteins modulated by CHQ. (i) CHQ accumulation in the lysosome inhibits the lysosome-autophagosome fusion and impairs degradation of proteins including the ubiquitin (Ub)-binding protein p62 and its binding partner pro-apoptotic LC3-II. (ii) CHQ intercalates into the DNA helix and cause relaxation of chromatin structure, which may be the mechanism of ClQ-mediated activation of a DNA damage-inducible kinase ATM. (iii) CHQ modulates activities of pleiotropic transcription factors p53 and NF-κB and may influence cross-talk between these pathways. (iv) p62 functional duality and positive p62 / NF-κB feedback loop. (v) Augmentation of pro-apoptotic activities of p53 may be a possible mechanism of CHQ-mediated radiosensitization. (vi) Autophagy inhibition by ClQ may counteract autophagic activation by TMZ and thereby sensitize glioma cells to chemotherapy.

The concurrent activation of cell death and pro-survival pathways through the modulation of autophagy might represent yet another death-survival axis regulated by CHQ: On the one hand, CHQ can activate cell death through the lysosome-initiated apoptosis via cathepsin signaling (63, 64). On the other hand, CHQ leads to the accumulation of a multifunctional protein chaperone p62 (also known as sequestome-1, SQSTM-1), whose expression is associated with increased cell proliferation, tumor growth and cytotoxic resistance in different types of human cancers (65). In gliomas, p62 expression correlates with the tumor grade and shorter survival (66, 67). As p62 is an autophagy adaptor targeted for degradation through autophagic clearance, autophagy inhibition by CHQ leads to the increase of the p62 protein levels (68). One of the mechanisms underlying pro-tumor activities of p62 relies on its ability to activate NF-κB, a key pathway regulating cell survival and proliferation. Augmented NF-κB signaling is linked to poor prognosis and treatment resistance in gliomas (69, 70). Moreover, there is evidence that activation of the p62/NF-κB signaling by CHQ may be further amplified through a positive feedback loop whereby CHQ-induced p62 activates NF-κB, which in turn activates the expression of p62 (71). Thus, inhibition of autophagy by CHQ can activate not only the lysosome-mitochondria death pathway, (63, 64) but also survival–promoting signaling mediated through the p62/NF-κB feedback loop (71). Considering that ATM is essential for the function of both p53 and NF-kB proteins, which often act in an antagonistic way in the regulation of cell survival, (72) and that CHQ modulates activities of all three factors (Figure 1), it is conceivable that p53 status is an important factor in determining cell fate in response to CHQ treatment.

The dichotomy of cellular responses elicited by CHQ is also manifest in its inhibitory effects on the inflammatory response which might be particularly important considering the tumor microenvironment. Normalization of the tumor vasculature has been implicated as a potential mechanism underlying the ability of CHQ to increase the efficacy of chemotherapeutic drugs, by facilitating their delivery to the tumor mass (73, 74). Indeed, there is evidence that CHQ normalizes the tumor vasculature through the reduction of vessel density, improvement of cell alignment, formation of tight junctions and promotion of quiescent phenotype of endothelial cells (73, 74). However, CHQ has also been shown to have pro-inflammatory effects in some types of cells. Within the CNS, CHQ inhibits pro–inflammatory cytokines in microglial cells, but not in astrocytes, in which it induces inflammatory cytokines through the activation of NF-κB signaling (46). Considering that GBs are tumors of astrocytic origin, their responses to CHQ may resemble those observed in astrocytes.

Thus, the ultimate outcome of CHQ treatment is likely to be determined by the intricate balance between activities of pleiotropic pathways involved in the regulation of autophagy, DDR and apoptosis/cell death (Figure 1).

## Chloroquine as potential anti-cancer drug: unsolved questions and confounding issues

Despite recent advances in the understanding of molecular mechanisms of anti-tumor effects of CHQ, a number of issues remain unsolved. One confounding factor is that experimental models used for investigating the effects of CHQ may not fully recapitulate distinctive characteristics of treatment-resistant GB. The current paradigm of therapeutic resistance in GB is centered on so-called glioma stem-like cells (GSCs). GSCs are considered the most clinically relevant type of glioma cells driving GBs propagation before and after therapy (75). It has been shown that GSCs possess an augmented DNA damage response (DDR), (76) which renders them capable of surviving cytotoxic treatments that are otherwise effective in killing non-stem glioma cells (76–78). In conjunction with augmented DDR, radiation-induced activation of anti-death and autophagic responses make important contributions to GSCs ability to escape from the cytotoxic effect of radiation (79, 80). Most of the existing studies addressing the effects of CHQ in glioma cells have used conventional serum-dependent cell lines that lack stemness properties and/or poorly recapitulate characteristic features of human GBs. For example, the human glioma cell line U87MG, which has been widely used as an experimental model for investigating biological responses mediated by CHQ (21, 22, 29, 30, 81, 82) does not reproduce certain characteristic traits of GBs such as an invasive tumor phenotype, intra-tumoral heterogeneity and high degree of intrinsic radio-resistance. Considering that GSCs are fundamentally distinct from non-stem glioma cells, it is conceivable that their responses to CHQ might also differ from those operating in the latter ones. Further underscoring this notion, activation of the p53 signaling by CHQ seems to lead to different outcomes in GSCs or non–stem glioma cells. In non-stem glioma cells with wtp53, p53-dependent apoptosis is a profound response to high concentrations of CHQ (≥20 μM) either applied alone or combined with other treatments (22, 29, 30). In contrast, GSCs with functional p53 do not activate apoptosis, but undergo predominantly a G_1_ arrest in response to similar CHQ concentrations (20).

Furthermore, the threshold of CHQ concentrations required for inducing cell death in the experimental setting (~20 μM) is considerably higher than clinically acceptable doses of CHQ (~5 μM). Therefore, the potential therapeutic value of clinically acceptable doses of CHQ for GB treatment requires further validation. Clarifying this question is particularly important considering the results of a phase I/II trial addressing the feasibility of dose escalation for CHQ treatment of GB (32). It was found that CHQ doses used for treating rheumatoid arthritis may not be sufficient to effectively inhibit autophagy when used in combination with TMZ and radiation in patients with GB (32). Likewise, CHQ potential in sensitizing glioma cells to radiation, observed under experimental conditions (single treatment with 10 Gy) (20) needs to be reproduced under clinically relevant conditions, applying hypofractionated radiation (multiple fractions of 2.0–2.5 Gy).

## Conclusion

The chemo- and radio-sensitizing effects of CHQ observed under experimental conditions warrant further explorations of CHQ potential as an adjuvant treatment for GB. In order to better define the potential benefits of using this drug as an adjuvant treatment for GB, the remarkable diversity of outcomes that can be elicited by CHQ need to be considered.

## Author contributions

PW: literature analysis, manuscript writing and preparation. SK: manuscript revision and preparation. EK: concept, literature analysis, manuscript writing, preparation and final approval.

### Conflict of interest statement

The authors declare that the research was conducted in the absence of any commercial or financial relationships that could be construed as a potential conflict of interest. The reviewer KR and the handling Editor declared their shared affiliation.
